# How many species of flowering plants are there?

**DOI:** 10.1098/rspb.2010.1004

**Published:** 2010-07-07

**Authors:** Lucas N. Joppa, David L. Roberts, Stuart L. Pimm

**Affiliations:** 1 Microsoft Research, 7 J J Thomson Avenue, Cambridge CB3 0FB, UK; 2 Durrell Institute of Conservation and Ecology, School of Anthropology and Conservation, University of Kent, Marlowe Building, Canterbury, Kent CT2 7NR, UK; 3 Royal Botanic Gardens, Kew, Richmond, Surrey TW9 3AB, UK; 4 Nicholas School of the Environment, Duke University, Box 90328, Durham, NC 27708, USA

**Keywords:** angiosperms, biodiversity hotspots, taxonomic effort, threatened species, total number of species, unknown species

## Abstract

We estimate the probable number of flowering plants. First, we apply a model that explicitly incorporates taxonomic effort over time to estimate the number of as-yet-unknown species. Second, we ask taxonomic experts their opinions on how many species are likely to be missing, on a family-by-family basis. The results are broadly comparable. We show that the current number of species should grow by between 10 and 20 per cent. There are, however, interesting discrepancies between expert and model estimates for some families, suggesting that our model does not always completely capture patterns of taxonomic activity. The as-yet-unknown species are probably similar to those taxonomists have described recently—overwhelmingly rare and local, and disproportionately in biodiversity hotspots, where there are high levels of habitat destruction.

## 1. Introduction

How many species there are in a taxon is an intrinsically interesting question (May [1–5]). It also has important implications for conservation. Recently discovered species are in biodiversity hotspots [6]—places with high levels of habitat destruction. As-yet-unknown species are likely to be in the same places and so in danger of extinction, if indeed they are found before they go extinct. Estimating how many such species there are is an essential step in setting conservation priorities.

There are two questions in estimating a taxon's total number of species. Surprisingly, the first is how many unique species taxonomists have already described. There are considerable uncertainties in the estimates of such species. Only when these are resolved can one ask the second question of how many more species there are that are presently unknown.

The first question is one of synonymy—taxonomists give different names to the same species inadvertently. There have been several recent estimates of the currently known number of unique species of plants [3,7–9], with the highest estimate twice the lowest one. [9] found a consistent percentage of synonyms within each family and, taking that rate of synonymy into account, estimated 352 282 unique flowering plant names.

We use the World Checklist of Selected Plant Families [10], a unique and continuously updated synonymized world list of plants that the Royal Botanic Gardens, Kew supplied. It has resolved problems of synonyms, but for only some plant families and around 110 000 species of seed plants. We use GrassBase, a similar list for the roughly 10 000 species of grasses [11].

We ask the second question for just the families in these synonymized checklists: how should one estimate the number of species remaining to be discovered? Previous estimates used scaling laws in food webs, abundance, body size, rarity and other methods to predict the total number of species in various taxa [1,2,12]. More recent attempts employ differing methods of extrapolation of the number of species described over time, with the expectation that the number of new species per time interval in a taxon will decline as the pool of unknown species diminishes [13,14]. Generally, they do not. In one study, New World grasses showed a consistent increase in the number of new species over time [15]! We shall show that this pattern is indeed a common one.

We find previous attempts wanting because none includes the number of taxonomists involved in describing species. The number of plant taxonomists active in any period (which we will define) has increased steadily over the 250 years of taxonomic history, a trend probably true of other taxa too. Not surprisingly, the raw number of species described over time has increased as well. By analogy to fishing statistics, one scales raw fish catches by the effort taken to acquire them to obtain ‘catch per unit effort’ as a measure of stock size. Here, we model the rate at which taxonomists ‘catch’ previously unknown species.

Our model has two factors. First, the greater the effort—the number of taxonomists involved in describing species—the more species they will describe in a given interval, other things being equal. We define ‘taxonomists’ simply as those who describe new species. Taxonomic effort is a powerful predictor of the number of species described.

Second, taxonomists have probably increased the efficiency of their efforts since the mid-1700s. That was when Linnaeus introduced the system of binomial nomenclature and founded modern taxonomic practice by providing as complete an account of all known species as he could. By ‘taxonomic efficiency’ we mean simply an increase in the number of species described per taxonomist, adjusted for the continually diminishing pool of as-yet-unknown species. Not all the taxonomists we polled (see below) thought taxonomic efficiency had increased. Were efficiency to have remained constant, the number of species described per taxonomist would decline continuously over time as the supply of undescribed species dwindled. We will show that for many taxa there is an increase in the number of species per taxonomist, typically for a century or so.

Finally, there are other confounding issues, also inspired by fishing analogies, to which we shall return.

## 2. An approach using taxonomic effort

The WCSP, together with GrassBase, present synonymized checklists of monocots, a monophyletic clade that includes approximately 20 per cent of all known flowering plants. These lists give a total count of 69 323 species of monocots. The WCSP checklist of the remaining flowering plants is less complete. We consider a total of 49 481 species that constitute less than a fifth of these non-monocot families.

For each 5-year interval, we calculate the number of unique species discovered and the number of taxonomists working. We expect the number of species described in interval *S*_i_ to depend on the number of taxonomists *T*_i_ actively describing species during that period,2.1
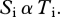


Our model consists of two elements. The first is the remaining number of species to be described, *S*_R_. It is the total number of species, *S*_T_, minus the cumulative number of species already described, ∑*S*_i_ up to the given year, *t*2.2
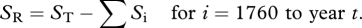


We chose 1760 as the start date to avoid the undue influence of Linnaeus's seminal work *Species plantarum* [16].

The second element is taxonomic efficiency, *E*. We assume that taxonomists have become more effective at finding and describing species now than in the past. For simplicity, we assume that this increase in efficiency increases linearly over time:2.3
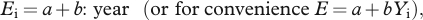
where *a* and *b* are estimated parameters. Efficiency need not increase, whereupon *b* would be zero. All things being equal, *S*_i_/*T*_i_ will decrease as the number of species still to be discovered declines. Also, *S*_i_/*T*_i_ will increase over time as efficiency increases, so the exact form will depend on the product of efficiency and species remaining,2.4
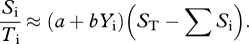


From this it follows that2.5
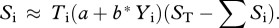


This is an intrinsically nonlinear statistical model, because there are four independent variables in the complete expression,2.6
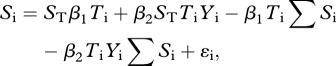
but only three parameters to be estimated: *S*_T_, *β*_1_ and *β*_2_, *ɛ*_i_ are the residuals.

The number of species described per period tends to be ‘spiky’, indicating the undue influence of monographs that describe many species in the year they appear followed by intervals when taxonomists described relatively fewer species. For obvious reasons, as the number of taxonomists increases, the influence of individual monographs declines and the relationship becomes smoother. To normalize the residuals, we took the logarithms of observed (*S*_i_) and predicted (*S*_T_ *β*_1_ *T*_i_ + *β*_2_ *S*_T_ *T*_i_ *Y*_i_−*β*_1_ *T*_i_ ∑*S*_i_−*β*_2_ *T*_i_*Y*_i_∑*S*_i_) numbers of species, and minimized the sums of squares of their differences. We used a grid search followed by a steepest-descent method to find values of the three parameters that minimized this sum of squares.

This logarithmic transformation creates large residuals when the numbers of species are very small, as they were in the mid-1700s. If at least 40 species had not been described by 1760, we started in the first 5-year period where the cumulative number of known species was 40 or more.

Our model does not permit estimates of confidence intervals based on parametric statistics. We can estimate the certainty of our estimates in two ways. First, we used a standard jack-knife procedure iteratively removing data from one 5-year interval at a time and successively returning the previously removed data. This procedure provided 47–50 different predicted total species estimates, depending on the taxon and the year in which the cumulative number of species was more than 40. We report their minima and maxima. Second, we re-ran the entire analysis using 10-year intervals, obtaining similar results to those reported here.

## 3. Results

### (a) Overall estimates of diversity

**Figure 1.**
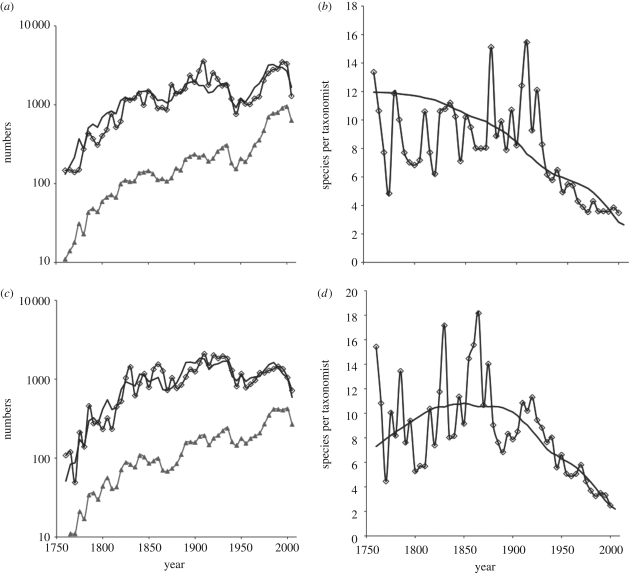
(*a*,*c*) Open diamonds are the logarithms of the number of species of monocot and (*b*) Selected non-monocot species described per 5-year interval against date. Filled triangles are the numbers of taxonomists active in describing species in each 5-year interval. Solid black lines are the models fitted to minimize the sums of squares of the differences between observed and predicted values. (*b*,*d*) Open diamonds are the ratios of numbers of species described per taxonomist against date. The solid black lines are model fits. (*a*,*b*) Monocot species; (*c*,*d*) non-monocot species.

For monocots (figure 1*a*), there is a broad increase in the number of species described per interval over time. The scale is logarithmic. The decline since 2005 represents incomplete data. Clearly, any method based simply on the number of species would conclude that there is no diminution of the pool of as-yet-unknown species. Figure 1*a* also shows the increasing number of taxonomists active in any period—essentially an exponential increase (linear on the figure's scale) since about 1800. There are dips in both numbers from the 1920s until the 1960s. Figure 1*b*,*d* shows the number of species described per taxonomist plotted on an arithmetic scale. These decline continuously over time.

For selected non-monocots, the number of species described per period increases until about 1850 and then remains roughly constant (figure 1*c*). The number of taxonomists again increases roughly exponentially. The number of species per taxonomist increases for about a century then declines steadily.

We estimate there should be an increase of 17 per cent in the number of species of monocots (range 13–18% using the jack-knife procedure; table 1). For the selected non-monocots in the database, the number of species should increase by 13 per cent (range 11–14% using the jack-knife procedure; table 1). These estimates broadly compare with [3], who independently arrived at an estimate of 20 per cent.

**Table 1.** Summary table of model results for all monocot families and selected non-monocots. Columns two and three list the number of currently known species present in the WCSP and GrassBase data, and the total number of species we estimate to exist. Columns four and five report the minimum and maximum number of species predicted using the jack-knife methodology see §3. Column six lists expert estimates of the total number of species. FTC indicates those families where the model did not converge on a number less than three times the current number of known species. Superscripts a–t denote the expert taxonomist that provided the estimate. Where the expert also provided a different number of currently known species we included that figure in column 2.

family	known species	total predicted	min	max	expert opinion	expert ratio
monocots total	69 323	80 901	78 573	81 879		
Orchidaceae	25 971	28 894	28 235	29 160	30 000^a^	1.16^a^
Poaceae	10 085^s^; 12 449^b^	11 445	11 264	11 513	13 000^c^	1.03^c^
Cyperaceae	5550	6225	6093	6295	5850–5950^d^; >6,150^e^	1.06^d^; 1.11^e^
Araceae	3081	5141	4502	5726	4000–4500^f^	1.46^f^
Bromeliaceae	3063	4108	3831	4358		
Asparagaceae	2733	4123	3862	4668		
Arecaceae	2406	2718	2650	2746	2,706^g^	1.12^g^
Iridaceae	2125	FTC	FTC	FTC	2,200^h^	1.04^h^
Alliaceae	2123	FTC	FTC	FTC		
Zingiberaceae	1516	1955	1846	2072	1,713^i^	1.13^i^
Eriocaulaceae	1206	2032	1836	2465		
Pandanaceae	1098	FTC	FTC	FTC		
Xanthorrhoeaceae	1083	FTC	FTC	FTC		
Liliaceae	716	1197	1105	3506		
Commelinaceae	710 700^j^	1003	935	2951	720–725^j^	1.04^j^
Dioscoreaceae	642	720	704	758		
Marantaceae	495 583^k^	642	583	728	636^k^	1.09^k^
non-monocots total	49 481	55 828	55 140	56 289		
Rubiaceae	13 072	18 787	17 691	19 727	16 000^t^	1.22^t^
Lamiaceae	7683	9400	9207	10 072		1.15–1.20^l^
Euphorbiaceae	6509	7793	7564	8088	7500^m^	1.2^m^
Myrtaceae	5668	8248	7718	9494		
Campanulaceae	2308	3064	2941	3246		
Phyllanthaceae	2021	4522	3770	FTC	<2500^m,n^	1.2^m,n^
Apocynaceae s.s.	1750	FTC	FTC	FTC	<2000^f^	1.14^o^
Begoniaceae	1485	2507	2190	2949	2000^g^	1.35^g^
Araliaceae	1432	2254	2004	2866		
Sapotaceae	1241	2728	2280	4243		1.10–1.15^p^
Fagaceae	1087	1713	1508	FTC	950^p^	1.06^q^
Verbenaceae	1015	FTC	FTC	FTC		
Bignoniaceae	825	FTC	FTC	FTC		
Oleaceae	684	FTC	FTC	FTC		
Chrysobalanaceae	531	FTC	FTC	FTC	600^r^	1.13^r^

^a^P. J. Cribb; ^b^B. Simon; ^c^R. Soreng; ^d^D. Simpson; ^e^W. Thomas; ^f^S. Mayo; ^g^A. Henderson; ^h^P. Goldblatt; ^i^J. Kress; ^j^R. B. Faden; ^k^H. Kennedy; ^l^A. Paton; ^m^P. Berry; ^n^K. Wurdack; ^o^D. Goyder; ^p^T. Pennington; ^q^P. Manos; ^r^G. Prance; ^s^[11]; ^t^[22].

### (b) Family-by-family results

**Figure 2.**
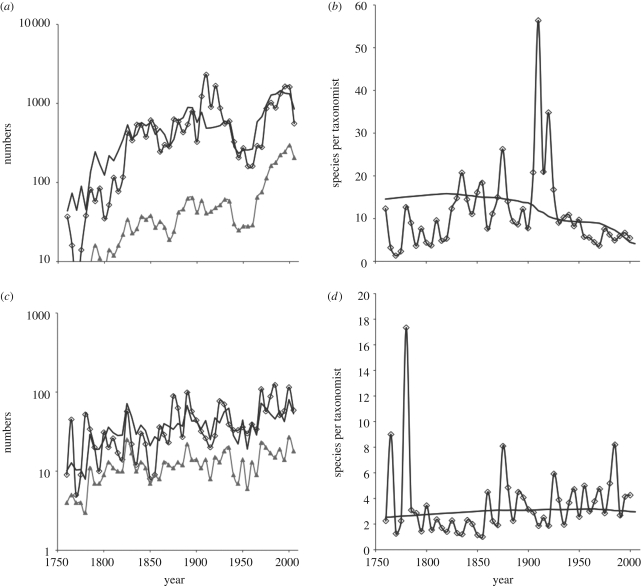
As figure 1, but for two selected families. (*a*,*b*) Orchids (Orchidaceae), show a century-long trend in declining numbers of species per taxonomist. (*c*,*d*) Irises (Iridaceae), in contrast, show a generally increasing number of species per taxonomist following early descriptions of species in the 18th century. Despite this, experts believe that almost all the species in this family will be described in the next five years.

We analysed individually all taxonomically complete families containing more than approximately 500 species. As an example, for orchids (figure 2*a*,*b*), the number of species per taxonomist increases very slightly then clearly decreases over time. The ‘spike’ represents the work of Rudolf Schlechter who, at his peak, described over 400 species per year between 1911 and 1913 [10,17].

For irises (figure 2*c*,*d*) in the late 1700s, large numbers of showy South African species were discovered and brought to Europe. Since 1800, the number of species per taxonomist has *increased* slowly and so our model does not provide a sensible estimate of the number of unknown species.

Table 1 shows the results for 17 taxonomically complete families of monocots presented in order of decreasing numbers of species. These families contain more than 93 per cent of all monocot species. Between 11 (Orchidaceae; range 9–12%) and 68 per cent (Eriocaulaceae; range 52–204%) more species remain to be discovered in each family.

We label the estimate for families where our estimate is more than three times the number of known species as ‘failing to converge.’ Four families did not provide sensible estimates.

There are 15 families in the WCSP database other than monocots that have more than 500 species (table 1), constituting 96 per cent of the species in the dataset we used. Ten of 15 families provided sensible estimates. The six families with the greatest numbers of species constitute 75 per cent of the species we model, and for them we predict increases from 20 per cent (Euphorbiaceae; range 16–24%) to more than twice the presently known number (Phyllanthaceae). These six families suggest a much higher number of unknown species than the 13 per cent we estimate for the group as a whole. That a subset of families provides different overall estimates than all families combined may seem contradictory, yet it reflects increasing specialization by taxonomists over time (see the electronic supplementary material).

### (c) How do our results compare with expert opinion?

In our second approach, we polled botanical colleagues for their estimates of how many species would eventually be described. We obtained estimates for 18 families this way (table 1). Their overall average—a 15 per cent increase in the present number of species—fits well with our model estimates. For three families, experts used a slightly different number of known species than in the catalogues we used above. For Poaceae, the expert provided a number of known species differing substantially from our tally.

For 11 of 18 families, expert opinion broadly matches the results of a quantitative modelling (table 1). In contrast, for three families (Iridaceae, Apocynaeae, and Chrysobalanaceae) where our estimates failed to provide sensible estimates, experts suggested that few species remain unknown (4%, 14% and 13%, respectively). How can we reconcile these opinions of few remaining unknown species with data showing either no decreases or sometimes even slight *increases* over time in the number of species described per taxonomist? By analogy to fishing catch-per-unit effort statistics, some families might have near-constant species per taxonomist ratios for decades—suggesting a large supply of unknown species—but then decline rapidly and unexpectedly as the ‘stock’ of such species is quickly exhausted.

Goldblatt justified his expectation that Iridaceae will be complete in about 5 years despite the generally *increasing* rate of species described per taxonomist over time (P. Goldblatt 2009, personal communication). The family is horticulturally desirable and has been deliberately targeted thoroughly in its known centres of diversity. Relatively poorly known areas, such as the wet tropics, hold few species. His work has been to revise genus after genus. He records that he is close to the end of genera that could be usefully revised and writes that ‘additions will just come to an abrupt end in the next 3–5 years.’ We will explore more complex models incorporating the taxonomic completion of subsets of plant families elsewhere.

## 4. Discussion

To summarize, the number of presently unknown plant species is thought to be 10 to 20 per cent of the number of known plant species. Approximately 13 per cent of the species in these synonymized data have been described since 1990. Of those, approximately 90 per cent are known from only one of the 300 or so regions into which the WCSP divides the world. Certainly, time may uncover other locations for these species, but that trend is balanced by the fact that, if the species were widespread, taxonomists would probably have found them earlier [18].

Overwhelmingly, the locations of these recent discoveries are critically imperilled—as are the species themselves ([19]; provides an exception). Of the species found since 1990 that occur in only one region, almost 80 per cent inhabit biodiversity hotspots [6]. These areas have many endemic species, by definition. Our results suggest that their numbers will increase further. Also by definition, these areas also have exceptionally high levels of habitat loss. Simply, unknown species are nearly all likely to be rare and in rapidly shrinking habitats, and hence likely to be deemed ‘threatened’ when taxonomists do describe them.

Brummitt *et al.* [20] suggest that 20 per cent of known plant species are threatened. If we take this estimate, then add to that our result that there are 10 to 20 per cent more unknown species that are also likely to be threatened, then 27 to 33 per cent of all plant species are probably threatened. These estimates are based on immediate threat, and do not consider further development of destructive factors—including climate disruption [21]—during the remainder of this century.
